# Pars plana Ahmed glaucoma valve implantation with triamcinolone-assisted vitrectomy in refractory glaucomas

**DOI:** 10.4103/0301-4738.67068

**Published:** 2010

**Authors:** Tanuj Dada, Shibal Bhartiya, Murugesan Vanathi, Anita Panda

**Affiliations:** Dr. Rajendra Prasad Centre for Ophthalmic Sciences, All India Institute of Medical Sciences, New Delhi, India

**Keywords:** Pars plana Ahmed glaucoma valve, refractory glaucoma, triamcinolone, vitrectomy

## Abstract

Glaucoma drainage devices are an option in refractory glaucomas for control of intraocular pressure (IOP). We evaluated the outcome of pars plana Ahmed glaucoma valve along with triamcinolone-assisted vitrectomy in 11 eyes with uncontrolled IOP on maximum tolerable antiglaucoma therapy. The mean preoperative IOP of 33.64 ± 5.99 (range 26 to 44 mmHg) decreased to 17.09 ± 2.26 (range 14 to 20 mmHg) and 17.45 ± 1.81mm of Hg (range 14 to 24 mmHg) at 6 and 12 months following surgery. The mean number of antiglaucoma medications decreased from 3.27 ± 0.05 to 0.64 ± 0.67 and 0.55 ± 0.6 at 6 and 12 months following surgery.

The use of pars plana tube shunts for surgical control of intraocular pressure (IOP) in refractory glaucomas such as glaucoma associated with aphakia, neovascularisation, post penetrating keratoplasty (PKP), and eyes with conjunctival scarring like, post-vitrectomy, and post traumatic glaucomas has already been established.[1–3]

The utility of triamcinolone-assisted vitrectomy has also been reported in different clinical situations.[4,5] We evaluated the use of pars plana Ahmed glaucoma valve (AGV) combined with triamcinolone-assisted vitrectomy in eyes with refractory glaucoma.

## Materials and Methods

Eleven eyes of 11 patients with uncontrolled intraocular pressure (IOP) on maximum tolerable antiglaucoma therapy (PKP glaucoma- 6, aniridia - 3, and aphakic glaucoma - 1, congenital glaucoma with aphakia and twice failed trabeculectomies - 1) who had undergone pars plana AGV (Model PC 7-10 eyes, Model PC 8 - 1 eye, New World Medical Inc, Rancho Cucamonga, CA, USA) implantation at our tertiary care center between March 2007 and April 2008 were evaluated in this retrospective study. Eyes with aniridia had undergone an additional lensectomy for subluxated cataractous lenses.

Clinical outcome assessment included IOP measurement with Tonopen XL, tube assessment with indirect ophthalmoscopy and anterior segment optical coherence tomography (ASOCT), corneal clarity, and any associated complications. Follow-up protocol was at weekly intervals in the first postoperative month and three-monthly thereafter for one year. Criteria for success were defined as follows: absolute success - IOP ≤ 18 mm Hg, qualified success - IOP < 18 mm Hg with ocular hypotensive medications.

Surgical Technique:Following peribulbar anesthesia, under surgical asepsis, a corneal traction suture was placed and a fornix-based conjunctival flap fashioned in the superotemporal quadrant.

After priming the AGV, the explant plate was sutured to the sclera, 10 mm from the limbus, with 8’0 monofilament nylon. The tube was then trimmed with the bevel up, to an intravitreal length of 6 mm and introduced into the vitreous via a sclerostomy incision 3.5 mm behind the limbus. A donor sclera patch was used to cover the tube at the site of the sclerostomy. The conjunctiva was then closed.

The anterior vitreous was first stained by injecting 0.1 ml of preservative-free triamcinolone acetonide (Aurocort, Aurolabs, Madurai, India; 4 mg / 0.1 ml) to help in its visualization, ensuring complete clearing of the area around the tube. An automated limited vitrectomy (cut rate - 500 and vacuum - 150 mm Hg) was then performed.

## Results

Mean age of the patients (all males) was 36.34 ± 20.07 years (range: 8 - 62 years) with a mean follow-up of 17.09 ± 6.17 months (range: 13-27 months). The mean preoperative IOP of 33.64 ± 5.99 (range: 26-44) decreased to 17.09 ± 2.26 (range 14 - 20) and 17.45 ± 1.81mm of Hg (range 14-24) at six and 12 months following surgery. The average number of ocular hypertensive medication decreased from 3.27 ± 0.05 to 0.64 ± 0.67 and 0.55 ± 0.6 at six and 12 months following surgery. Absolute and qualified success was noted in five and nine out of 11cases at six months and six and nine out of 11 cases at 12 months after surgery, respectively [Table 1].

**Table 1:** Intraocular pressure control in the various indications for Ahmed Glaucoma Valve (AGV)

Age (years)	Indication for AGV	Intraocular pressure (with number of antiglaucoma drugs)		
		Preoperative (mm Hg)	At 6 months follow-up (mm Hg)	At 12 months follow-up (mm Hg)
17	Aniridia wih subluxated cataractous lens with secondary glaucoma	32 (3)	16 (0)	16 (0)
15	Aniridia wih subluxated cataractous lens with secondary glaucoma	28 (3)	16 (0)	14 (0)
8	Twice failed trabeculectomy for congenital glaucoma with aphakia	38 (3)	16 (0)	16 (0)
14	Aniridia with secondary glaucoma with aphakia	42 (4)	18 (1)	18 (1)
42	Post-PKP glaucoma	28 (4)	18 (0)	18 (0)
50	Post-PKP glaucoma	36 (3)	20 (2)	24 (2)
56	Aphakic glaucoma	44 (3)	20 (1)	18 (1)
48	Post-PKP glaucoma	30 (4)	20 (1)	20 (1)
62	Post-PKP glaucoma	26 (3)	16(0)	16 (0)
46	Post-PKP glaucoma	36 (3)	14 (1)	18 (1)
50	Post-PKP glaucoma	30 (3)	14 (1)	18 (1)

IOP: Intraocular pressure, PKP: Penetrating keratoplasty

**Figure 1:**
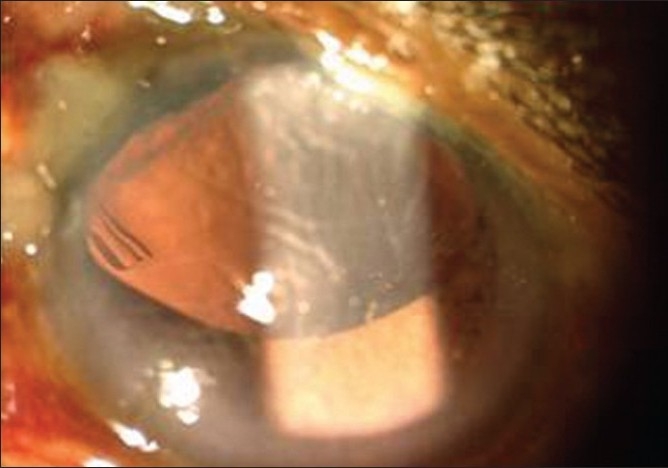
Postoperative clinical picture with pars plana Ahmed glaucoma valve (AGV) tube in the vitreous cavity

**Figure 2:**
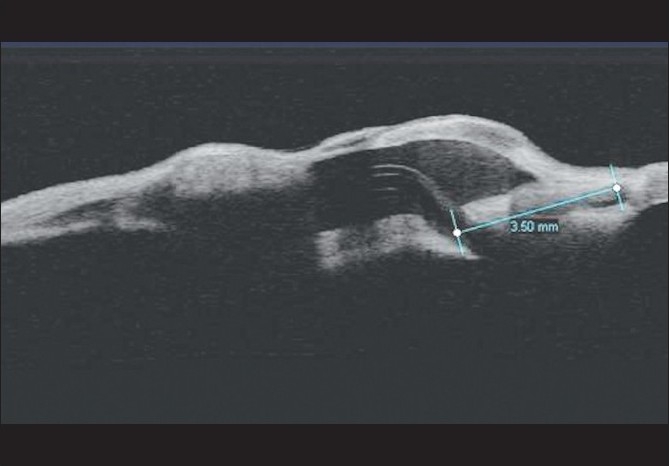
Anterior segment optical coherence tomography (ASOCT)picture showing tube entry via sclerostomy 3.5mm behind the limbus

All eyes maintained a patent tube with no vitreous blockage as visualized on slit-lamp biomicroscopy and ASOCT [Figs. 1 and 2]. One patient with post-PK glaucoma (case number 6) developed a graft infection (culture-proven *Staphylococcus epidermidis*) six months after surgery which was vigorously treated with antimicrobial therapy (cefazolin 5%, and tobramycin 1.3% eye drops). None of the patients were observed to have any retinal complications at the last follow-up.

## Discussion

We achieved an absolute and qualified success of IOP control in 5/11 and 9/11 eyes at 6 months; 6/11 and 9/11 eyes at 12 months after surgery, respectively. Transient hypotony occurred in three patients in the early postoperative period which resolved on medical therapy (systemic prednisolone 1 mg/kg, topical 1% prednisolone acetate drops one-hourly, and 1% atropine sulphate ointment thrice daily) by six weeks. None of the operated eyes had any vitreous blockage of the tube, demonstrating the efficacy of the triamcinolone-assisted vitrectomy. Triamcinolone aids visualization of the vitreous and ensures its complete removal from around the tube, helps in establishing tube patency intraoperatively and also decreases the postoperative inflammation associated with the procedure.

Schlote *et al*. reported an IOP control of 21 mmHg or less in ten of 11 eyes (91%) of secondary glaucoma at one year following pars plana valve surgery. Complications included transient hypotony, transient choroidal effusion and an intermediate increase in IOP.[1] Faghi *et al*. found the combination of pars plana vitrectomy (PPV) and panretinal photocoagulation to be efficacious and safe in patients with neovascular glaucoma and vitreous hemorrhage.[2]

Sidoti *et al*. analyzed 34 eyes undergoing pars plana tube shunt insertion before, concurrent with, or after PK and found that 12 and 24 months’ life-table rates for complete success after both glaucoma drainage device implant and PK were 63% and 33%, respectively. Twelve and 24-month life-table success rates for IOP control and corneal graft clarity were 85% and 62%, and 64% and 41%, respectively.[6]

The mean percentage decrease in corneal endothelial cell density in subject eyes has been reported to be 3.5% at one month, 7.6% at six months and 10.5% at 12 months after surgery with the greatest decrease being closest to the tube, following limbal AGV implantation.[7] Lee *et al*. also corroborated this, reporting a 22.6% decrease in endothelial cell density, 24 months after surgery in the supratemporal area, the site closest to the tube. The average endothelial cell loss increased with time: 15.3% at 12 months and 18.6% at 24 months after surgery.[8]

In our case series, graft remained clear in five out of the six PKP patients (83.3%) at one year following surgery, with one graft failure occurring due to graft infection. Our case series demonstrates that pars plana insertion of AGV combined with triamcinolone-assisted vitrectomy results in good IOP control, with no corneal decompensation.

Pars plana AGV implantation combined with triamcinolone-assisted vitrectomy is a useful therapeutic option for refractory glaucomas with aphakia, especially in eyes with compromised endothelial counts.
